# Dynamic geospatial modeling of mycotoxin contamination of corn in Illinois: unveiling critical factors and predictive insights with machine learning

**DOI:** 10.3389/fmicb.2023.1283127

**Published:** 2023-11-01

**Authors:** Lina Castano-Duque, Edwin Winzeler, Joshua M. Blackstock, Cheng Liu, Noemi Vergopolan, Marlous Focker, Kristin Barnett, Phillip Ray Owens, H. J. van der Fels-Klerx, Martha M. Vaughan, Kanniah Rajasekaran

**Affiliations:** ^1^Food and Feed Safety Research Unit, Southern Regional Research Center, Agriculture Research Service, United States Department of Agriculture, New Orleans, LA, United States; ^2^Dale Bumpers Small Farms Research Center, Agriculture Research Service, United States Department of Agriculture, Booneville, AR, United States; ^3^Microbiology and Agrochains Wageningen Food Safety Research, Wageningen, Netherlands; ^4^Atmospheric and Ocean Science Program, Princeton University, Princeton, NJ, United States; ^5^Agricultural Products Inspection, Illinois Department of Agriculture, Springfield, IL, United States; ^6^Mycotoxin Prevention and Applied Microbiology Research Unit, United States Department of Agriculture, Agricultural Research Service, National Center for Agricultural Utilization Research, Peoria, IL, United States

**Keywords:** *Aspergillus*, *Fusarium*, machine learning, gradient boosting, neural network, aflatoxin, fumonisin, soil

## Abstract

Mycotoxin contamination of corn is a pervasive problem that negatively impacts human and animal health and causes economic losses to the agricultural industry worldwide. Historical aflatoxin (AFL) and fumonisin (FUM) mycotoxin contamination data of corn, daily weather data, satellite data, dynamic geospatial soil properties, and land usage parameters were modeled to identify factors significantly contributing to the outbreaks of mycotoxin contamination of corn grown in Illinois (IL), AFL >20 ppb, and FUM >5 ppm. Two methods were used: a gradient boosting machine (GBM) and a neural network (NN). Both the GBM and NN models were dynamic at a state-county geospatial level because they used GPS coordinates of the counties linked to soil properties. GBM identified temperature and precipitation prior to sowing as significant influential factors contributing to high AFL and FUM contamination. AFL-GBM showed that a higher aflatoxin risk index (ARI) in January, March, July, and November led to higher AFL contamination in the southern regions of IL. Higher values of corn-specific normalized difference vegetation index (NDVI) in July led to lower AFL contamination in Central and Southern IL, while higher wheat-specific NDVI values in February led to higher AFL. FUM-GBM showed that temperature in July and October, precipitation in February, and NDVI values in March are positively correlated with high contamination throughout IL. Furthermore, the dynamic geospatial models showed that soil characteristics were correlated with AFL and FUM contamination. Greater calcium carbonate content in soil was negatively correlated with AFL contamination, which was noticeable in Southern IL. Greater soil moisture and available water-holding capacity throughout Southern IL were positively correlated with high FUM contamination. The higher clay percentage in the northeastern areas of IL negatively correlated with FUM contamination. NN models showed high class-specific performance for 1-year predictive validation for AFL (73%) and FUM (85%), highlighting their accuracy for annual mycotoxin prediction. Our models revealed that soil, NDVI, year-specific weekly average precipitation, and temperature were the most important factors that correlated with mycotoxin contamination. These findings serve as reliable guidelines for future modeling efforts to identify novel data inputs for the prediction of AFL and FUM outbreaks and potential farm-level management practices.

## 1. Introduction

Mycotoxin contamination of food and feed crops, such as corn, in the United States (US) leads to annual losses estimated in the range of $418 million to $1.66 billion (Vardon et al., 2003; Wu, 2006; Mitchell et al., 2016; Johns et al., 2022). These toxins are produced by distinct fungal species of *Aspergillus* and *Fusarium* (Proctor et al., 2018; Munkvold et al., 2019) and are well characterized by their severe health impacts on humans, livestock, and animals (Vardon et al., 2003). In corn, aflatoxins (AFL) are primarily caused by *A. flavus* and *A. parasiticus*, and fumonisins (FUM) are produced by *F. verticillioides* and *F. proliferatum* (Woloshuk and Shim, 2013). The severity of disease epidemics and associated mycotoxin contamination is driven by environmental and agronomical conditions, with the former being the most relevant. Each fungal species has optimal conditions that favor inoculum formation and dispersal, disease development, and mycotoxin production. For example, for *A. flavus*, the optimal growth temperatures are 30 to 35°C (Abdel-Hadi et al., 2012), while for *F. verticillioides*, temperature ranges between 20 and 25°C (Medina et al., 2014) are ideal for growth. These temperature optima tend to favor the growth of aflatoxin-producing fungal species such as *Aspergillus* spp. in southern latitudes (Klich, 2002; Kerry et al., 2021), whereas these optima favor the growth of *Fusarium* spp. in northern latitudes (Zingales et al., 2022). The geographical distribution of these fungi in the US will most likely change due to climate change leading to an expansion of the temporal and latitudinal temperature growth optima of these mycotoxigenic fungi (Nnadi and Carter, 2021; Yu et al., 2022; Zingales et al., 2022). Additionally, in general, hot conditions favor *A. flavus* conidiation and dispersal and *Fusarium* infection of corn (McMillian et al., 1985; Payne et al., 1986, 1988; Diener et al., 1987; Widstrom et al., 1990; Payne and Widstrom, 1992; Guo et al., 1996; Sétamou et al., 1997; Scheidegger and Payne, 2003; Cotty and Jaime-Garcia, 2007). Furthermore, kernel water activity (0.92–0.97 aw) and insect injury contribute to mycotoxin production (Warfield Colleen and Gilchrist David, 1999; Miller, 2001; Munkvold, 2003; Bush et al., 2004).

The agroecosystem and production of fungal mycotoxins involve soil biogeochemical characteristics that impact both crops and fungi. For example, carbonate content, saturated hydraulic conductivity, pH, bulk density, and texture, among others, influence soil microbial communities, and which species of fungi, including pathogens, might dominate in a given soil environment (Weil and Brady, 2002). Soil property variability is related to factors such as geologic deposition, age of material, climate, duration of soil development, land use history, and other factors. Much of the soils in Northern IL were formed in glacial and periglacial deposits from the Wisconsin glacial episode of the late Pleistocene associated with various advances and retreats of the continental Laurentide ice sheet, while soils in the more southern portion of the state developed from older parent materials with a difference in soil age of approximately 100,000 years or more (Bergstrom et al., 1976). The soil in Northern IL is richer in clay and carbonate materials. Previous studies in IL have shown a north–south difference in historical mycotoxin contamination (Castano-Duque et al., 2022). The 40° latitude in IL marks a geographical and meteorological boundary in the state that leads to differences in weather from mixed humid to wintry conditions (Köppen, 2011; Beck et al., 2018). Correspondingly, warmer, more humid conditions in Southern IL, provide relatively more favorable conditions for the fungi to infect corn and produce mycotoxins at lower latitudes compared to higher latitudes.

Integrated pest and disease management strategies that aim to minimize economic losses associated with mycotoxin contamination need to account for spatial and temporal-specific factors as well as weather, crop health and genetics, past agronomic practices, and soil characteristics. Mycotoxin contamination assessment tools are key for growers and stakeholders to be informed of the potential probabilities of mycotoxin outbreaks and facilitate the deployment of strategies aimed at controlling disease and grain contamination (Focker et al., 2020). Satellite-acquired databases, including the normalized difference vegetation index (NDVI), have been previously used in studies evaluating mycotoxins in European wheat and led to higher mycotoxin model accuracy (Wang et al., 2022). Several studies have shown significant correlations among climate, soil properties, NDVI, and agricultural management practices with AFL contamination in Africa, as reviewed by Keller et al. (2022). AFL studies conducted in Eastern and Western Kenya (Mutiga et al., 2014) support the utility of remote-sensing data such as NDVI, rainfall, and soil properties (such as organic carbon content, pH, total exchangeable bases, salinity, texture, and soil type) to accurately predict AFL contamination (Smith et al., 2016). Soils and their interactions with hydroclimate are highly variable (Smith et al., 2019; Vergopolan et al., 2022) and influence fungal and microbial populations in soils.

Predicting mycotoxin contamination through models has benefited corn production in other countries, but US corn growers, which produce the most corn in the world, have yet to realize such benefits. Published models assess mycotoxin contamination of milk in Eastern Europe (Van der Fels-Klerx et al., 2019) and mycotoxin risk in small grains, corn, and other cereal crops in Italy (Leggieri et al., 2021), Serbia (Liu et al., 2021), Europe (Wang et al., 2022), and Korea (Lee et al., 2018). Machine learning (ML) technologies such as artificial neural networks have been developed in Europe to identify mycotoxin contamination in corn kernels post-harvest using electronic nose technology in northern Italy (Leggieri et al., 2021). Mycotoxin contamination prediction modeling involves other factors, such as fungal development and dispersal. Spore dispersal modeling is an actively developing field of research worldwide, as reviewed by González-Domínguez et al. (2023). Furthermore, the impact of AFL predictive risk models in sub-Saharan Africa has been reviewed by Keller et al. (2022), showing the economic impact of these technologies. The basis of these models served as a general roadmap to model the prediction of AFL and FUM contamination in IL-US. In the US, modeling of fungal diseases has been done by the *Fusarium* head blight (FHB) risk assessment tool from the US Wheat and Barley Scab Initiative (https://www.wheatscab.psu.edu/) that uses modeling approaches to alert wheat and barley growers of FHB risk (Shah et al., 2018). A US-based predictive model based on insurance claims used as a proxy for mycotoxin contamination values is available for corn (Yu et al., 2020, 2022). However, insurance claims are not necessarily equivalent to actual mycotoxin values. Previous US models targeted the prediction of multinomial levels of AFL and FUM contamination and showed that ML approaches can detect weather factors pre-harvest that significantly impact mycotoxin accumulation in IL (Castano-Duque et al., 2022).

The need for US-focused models is even more important when considering that mycotoxin contamination is predicted to increase in the years to come due to climate change (Van der Fels-Klerx et al., 2016, 2019). Climate change models predict increased fungal growth and subsequent toxin production (Battilani et al., 2016) due to negative effects on crop host health and the interaction of multiple abiotic factors such as drought, leading to losses in corn production (Long Stephen et al., 2006; Battilani et al., 2016; Peng et al., 2020). Increasing temperatures, longer drought periods, and rising carbon dioxide levels (Vaughan et al., 2014, 2016) as the effects of climate change are expected to continue leading to increased crop vulnerability combined with favorable conditions for fungi such as *Aspergillus* spp. to grow and produce toxins. These conditions will lead to an increase in plant stress hormones that have been shown to alter plant defense responses (Guo et al., 2008), making the crop more vulnerable to fungal infection and mycotoxin contamination. Therefore, it is predicted that climate change has the potential to negatively impact northern corn-producing states (Corn Belt), as projected weather conditions will increase AFL-related risk (Wu et al., 2011). Risk assessment analysis using 16 climatic models from 2031 to 2040 in the US has shown that 89.5% of corn-growing counties will experience an increase in AFL prevalence, and this projection includes corn in the northern states, suggesting the spread of fungal infection and mycotoxin contamination from southern regions toward the northern Corn Belt (Yu et al., 2022), where it has the potential to be even more devastating to corn production. Models developed by using mycotoxin data from major US corn-growing states and trained using weather, satellite, and geographically dynamic features for US-focused areas are critically needed. They will benefit corn growers by mitigating losses projected to occur in extreme weather events associated with climate change.

We developed a mycotoxin model that is geospatial dynamic and IL-focused to identify novel risk factors correlated with mycotoxin contamination, we applied machine learning predictive models. We provided new information about the influence of soil properties and remote-sense data (NDVI) on the prediction of AFL and FUM contamination in corn. Understanding the correlations of these factors with mycotoxin contamination can lead to region time-specific targeted integrated pest management practices to prevent AFL and FUM outbreaks, thus serving as guidelines to identify factors that contribute to the contamination risk.

## 2. Materials and methods

### 2.1. Mycotoxin data

We used 14 years of historical contamination data, daily county-specific weather data, remotely sensed satellite reflectance data, and data on soil-specific features. Feature engineering (Battilani et al., 2013; Van der Fels-Klerx et al., 2019) was used in combination with gradient boosting models (GBMs) (Friedman, 2001) and neural networks (NNs) to predict (Torgo, 2016) AFL and FUM. In this study, we used 1,772 observations of mycotoxin concentrations, AFL, and FUM from IL-grown corn. Data on the response variable were obtained from historic yearly survey summaries for 2003–2004 and 2008–2021 (data for 2020 were not available due to the COVID-19 pandemic) published by the IL Department of Agriculture. The years used for model validation were selected from these data based on the percentage of incidence of high contamination events. AFL was measured in 2010 (960 data points, 4% incidence of high contamination levels) and FUM was measured in 2016 (960 data points, 8.1% incidence of high contamination levels). The sample collection was done as described by Castano-Duque et al. (2022); briefly, the kernel sample collection was performed at harvest by randomly collecting 2.3–4.5 kg of whole kernels from four corn producers from each county. While mycotoxin analyses were conducted as soon as possible after collection, to minimize post-harvest mycotoxin accumulation, the samples were dried and stored at 4°C. This mycotoxin data set was zero-inflated, with high contamination levels being rare events (Castano-Duque et al., 2022). This issue was considered during data preparation for ML analysis by using the synthetic minority oversampling technique (Torgo, 2016) that allowed us to generate a balanced data set of the high and low contamination events.

### 2.2. Output variables and correlation analysis

We categorized into discrete variables the AFL and FUM contamination values by following the US Food and Drug Administration's legal threshold limits for mycotoxins in feed (https://www.fda.gov/food/natural-toxins-food/mycotoxins). For AFL, a high category was considered for concentrations >20 ppb (20 ng/g) and low for levels 20 ppb or lower (Supplementary material 1). For FUM, a high contamination level was for concentrations >5 ppm (5,000 ng/g), and the rest of the observations were low (Supplementary material 1). The FUM threshold used is the FDA guidance for equids and rabbits, which is lower than for other classes of livestock. A correlation analysis was performed among all the predictors and output variables using a confidence level of 0.95 for the correlation and clustering method in R (Team, 2017).

### 2.3. Weather, satellite, and soil data

Soil physical and chemical properties, satellite reflectance data, and soil moisture were analyzed to account for geographic, geomorphic, and surficial geological heterogeneity relating to crop health and soil habitat suitability for mycotoxin-producing fungi. All the satellite data were extracted and summarized by the county after refining them to reflect only land uses for corn and wheat (Boryan et al., 2011). For the extraction of crop specificity of satellite data, a data mask layer was generated for corn acreage for each year of study from the National Agricultural Statistics Service Cropland Data layer (NASS CDL—https://nassgeodata.gmu.edu/CropScape/) data set (Boryan et al., 2011). NASS CDL provides 30-meter grid cell estimates of crop type grown for the Conterminous United States for each year starting in 2005 to the present. Each grid cell represents a best estimate of crop type for a 30-meter by 30-meter division of land. To make the data mask, in each year of study, for each grid cell classed as corn in Illinois, a value of 1 was assigned, while a value of 0 was assigned for all other land uses. Each time-series normalized difference vegetation index (NDVI) layer from the MODIS satellite data was multiplied by the mask so that only pixels of MODIS with their centroids falling within corn acreage were tabulated for the county-level NDVI averages (Didan, 2021). The same process was repeated for wheat, with a separate mask file produced from the NASS CDL and separate NDVI county-level averages produced.

Soil properties were similarly aggregated to the county level by using the NASS CDL data mask. In this case, values of 1 were assigned to cropland, and values of 0 were assigned to other land uses such as forest or urban (Boryan et al., 2011). Soil properties were then queried for all pixels with a value >0 in the Soil Properties data layer from UC Davis and USDA NRCS (Walkinshaw et al., 2022). Mean values for all soil properties for cropland were tabulated to represent cropland soil properties by county. Climatological soil moisture estimates were obtained for the top 5 cm of the soil in IL from SMAP-HydroBlocks at 30 m resolution, developed by combining microwave satellite remote sensing, radiative transfer modeling, machine learning, and a high-resolution land surface model (Vergopolan et al., 2020, 2021).

The physical soil properties available for examination within the mycotoxin model context were water-holding capacity, hydraulic conductivity, bulk density, and soil texture (sand, silt, and clay content). Soil chemical properties include calcium carbonate content, cation exchange capacity, electrical conductivity, pH, and organic matter content. Estimates were determined for the 800 × 800 m pixels for various soil depth increments using measured values and interpolation techniques (Walkinshaw et al., 2022). The soil data were queried for soil properties by examining the intersection of each pixel with the cropland reported in the national cropland data layer of the National Agricultural Statistics Service of the USDA (Boryan et al., 2011). This national cropland data layer (CropScape—https://nassgeodata.gmu.edu/CropScape/) provides estimates of cropping land use for 30 m × 30 m land units (pixels) throughout the contiguous continental US for a 5-year summary period prior to the publication date (the summary period used was 2017–2021). For each county, the cropland was characterized for the final 5 years of the study. Only the land area that was planted in crops for the period of study was summarized for its soil properties, which were aggregated to the county-wide level as mean soil property estimates. These estimates excluded urban, forested, and other land uses not pertaining to crop production.

Soil moisture estimates (m^3^ H2O/m^3^-soil) were extracted from the SMAP-HydroBlocks (Vergopolan et al., 2021) 2015 to 2019 soil moisture average. SMAP-HydroBlocks is a satellite-based surface soil moisture product available at 30 m resolution over the conterminous United States. SMAP-HydroBlocks is derived from NASA's Soil Moisture Active-Passive product and the HydroBlocks-Radiative Transfer Model using a “cluster-based merging scheme” (Vergopolan et al., 2020) that is parameterized using machine learning-guided refinements based on relations among other *in situ*, satellite-based observation, and land use-land cover characteristics (Vergopolan et al., 2020). The data product provides soil moisture estimates for the top 5 cm of the soil surface at 6-h intervals. In turn, SMAP-HydroBlocks is sensitive to rainfall, drought, and evapotranspiration dynamics relevant to the microenvironment of mycotoxin-producing fungi.

Historical daily average air temperature, precipitation, and general vegetation index data were obtained from GRO-Intelligence (https://gro-intelligence.com/). A non–crop-specific vegetative index from GRO-intelligence was calculated from satellite data by taking into consideration multiple light spectra to enhance the presence of green vegetation by calculating the normalized difference vegetation index (NDVI), and this NDVI will be called veg_index to differentiate it from crop-specific NDVI. Crop-specific NDVI was calculated for each pixel of reported corn production for each year studied and for each pixel of reported winter wheat production for each year studied. The NDVI values, which were identical to the pixels of soil property estimates, were also summarized with a county-wide mean to characterize NDVI for corn and wheat-cropped land over time. NDVI was calculated from the MODIS satellite data (Didan, 2021) by obtaining the difference between the near-infrared reflectance and the red reflectance divided by the sum of the near-infrared and the red reflectance given in the MODIS data set. Higher values of NDVI are given when vegetation is healthy and green, as it absorbs red light and reflects near-infrared light. These NDVI values are available every 16 days from the MODIS satellite from February 2000 to 2023 and are reported in a 250-m resolution. We extracted these time-series values from MODIS data for the pixels determined to be growing either corn or wheat for each 16-day period available using Google Earth Engine. The crop-specific NDVI and veg_index (general NDVI) data were then interpolated into weekly summaries for the periods of study.

Historic meteorological data, soil properties data, and NDVI data were linked to the county-level mycotoxin data by using the county and the year as common information. After linking toxin data with weather, NDVI, and soil data, there were 1,772 data points across 99 counties.

### 2.4. Features engineering and data imputation for the AFL modeling

Daily average precipitation (mm) and air temperature (°C) data, averaged per county for the 14 years of historical data, were acquired from GRO-Intelligence. Furthermore, we used the geographical centroids of each county (latitude and longitude) and the climate zones (Friedman, 2001). For AFL models, we engineered a weekly aflatoxin risk index (ARI) as the cumulative sum per week of daily ARI. The monthly ARI was previously described (Castano-Duque et al., 2022); herein, we modified it to calculate a daily ARI (Eq. 1). These ARI equations have been applied to data from Eastern Europe in particular from Ukraine, which has a climate equivalent to IL (Van der Fels-Klerx et al., 2019).


(1) $$\begin{array}{c} \text{ARI= growth or weighted}\_\text{growth }\times \text{ afla or weighted}\_\text{afla }\times \\ \text{dispersal }\times \;(\text{1 + ECB}\_\text{damage}) \end{array}$$

In the daily ARI, fungal growth was calculated on a daily basis (Battilani et al., 2013) as described by Castano-Duque et al. (2022). Weighted fungal growth (10% of original growth) was generated for days of the month throughout the year when there was no corn in the field. The distribution of mycotoxin levels in relation to the thermal climate zone in IL (Friedman, 2001) has been examined and linked to differences in mycotoxin contamination distribution (Castano-Duque et al., 2022). In IL, there are two climate zones: a mixed-humid climate zone with a monthly outdoor temperature below 7°C during the winter and a cold climate zone with 5,900 heating degree days per year (18.3°C basis) (Baechler and Love, 2004). These climatological differences were considered in the model by assuming that the planting date in the mixed-humid zone started about 15th May and in the cold zone in April. This was assumed to be different in the cold and mixed-humid counties due to differences in no-corn in the field times in northern and southern states (January to April, 15th May, and December for cold and January to April and December for mixed-humid). These dates were included by calculating the ARI (Eq. 1). One of the assumptions in our model is that during days of the year when there is no corn in the field, the fungal growth was 90% less. The daily AFL production index was calculated (Battilani et al., 2013) as described by Castano-Duque and Vaughan (Castano-Duque et al., 2022). The spore dispersal was set as an ON/OFF switch (Van der Fels-Klerx et al., 2019; Castano-Duque et al., 2022). The assumption in our model is that if there is any daily precipitation, the dispersal is OFF, and if there is no daily precipitation, the dispersal is ON. Wind and other variables are involved in the spore dispersal of *Aspergillus* (Segers et al., 2023) and *Fusarium* (Hoffmann et al., 2021) species; nevertheless, only precipitation was used in this model due to the consistency of daily historical records throughout the geographical region studied in this case study. Insect damage was calculated for European corn borer damage by using growing degree days (Tbase = 6C and Tcut = 30°C) and the logistic equation (Maiorano et al., 2009) (Eq. 2).

A = 7.3
Um = 0.013
Lambda = 1,236.913
R^2^ = 0.718
e = exp(1)

(2) $$\begin{array}{r} \text{ECB}\_\text{damage = A }\times \text{ exp}\{-\text{exp}[(\mu_{\text{m}}\text{ x e/A})\;\times \\ \left(\text{lambda }-\text{ cumGDD + 1}]\right\} \end{array}$$

where ECB (European corn borer) represents damage to the ear by insects, *A* represents the asymptotic maximum damage level observed in the field approximated to the higher first decimal, μ_m_ represents the maximum specific damage rate, λ represents the intersection of the tangent in the inflection point with the *x*-axis, and accGDD represents the accumulated GDD. Both μ_m_ and λ are estimated parameters in Maiorano and Reyneri (Maiorano et al., 2009).

For any missing values in the weekly ARI or vegetative index features, we performed imputation using multivariate imputation by chained equations; in specific, the predictive mean matching (PMM) method was used in R (van Buuren and Groothuis-Oudshoorn, 2011; Team, 2017). This imputation method allowed us to determine plausible data values by adding a data point from the original data whose regression-predicted values are closest to the regression-predicted value for the missing data point from the simulated regression model (van Buuren and Groothuis-Oudshoorn, 2011). Due to the high number of missing data for ARI in weeks 8, 16, 24, 32, 40, and 48, we decided to remove these input features from the model. Finally, we linked the AFL data to the feature data set to create a set of 1,772 data points and 153 features or predictors. The inputs for the AFL model were weekly ARI for each county (Supplementary Table 1). We also added to our input features an average bi-weekly NDVI per county during the weeks of the month when corn might not be in the field (below week 13 and above week 45); an average corn and wheat NDVI; soil properties, latitude, and longitude.

### 2.5. Weather data and imputation for the FUM modeling

For FUM modeling, we could not use feature engineering functions such as the ARI calculated for AFL produced by *Aspergillus* because these equations were generated using *Aspergillus* spp growth curves. The inputs for the FUM model were weekly average temperature and precipitation. We also added to our input features an average bi-weekly veg_index per county during the days of the month when there is no corn in the fields; an average corn and wheat-specific NDVI; soil properties; latitude; and longitude. The daily precipitation and daily average temperature data were averaged weekly per county; each calendar year of weather data was kept separate and linked to mycotoxin yearly data for the 14 years of historical data per year and county (Supplementary Table 1). Average temperatures were expressed in degrees Celsius, and each county was assigned its geographical centroids (latitude and longitude), climate zones (Beck et al., 2018), crop-specific NDVI, and veg_index. For any missing values in our weekly weather data, crop-specific NDVI, and veg_index features, we performed imputation using multivariate imputation by chained equations, specifically the predictive mean matching (PMM) method in R (van Buuren and Groothuis-Oudshoorn, 2011; Team, 2017). Due to the high number of missing data for weather variables in weeks 8, 16, 24, 32, 40, and 48, we decided to remove these input features from the model. Finally, we linked the FUM data to the feature data set to create a set of 1,772 data points and 199 features or predictors.

### 2.6. Gradient boosting machine learning

GBM was used to perform the prediction of mycotoxin contamination, as GBM allows for the determination of the relative influence of input features on the output variable. The GBM software package in R was used. This software provided extensions to Freund and Schapire's AdaBoost algorithm and Friedman's gradient boosting machine (Friedman, 2001). For performing GBM, we first removed the IL county identifier from the data set and the data for the year that was used for validation, as these would lead to overfitting the model. Next, data were balanced by using the synthetic minority oversampling technique (SMOTE) from the DMwr package in R (Team, 2017), which created a new balanced data set by oversampling observations from the high contamination level class. Finally, the balanced data were partitioned to generate training and testing sets by using a 70 to 30 ratio (Supplementary material 1). We performed GBM for AFL and FUM separately, using the following flags on the training data: a threshold of 500 trees, an interaction depth of 1, a shrinkage of 0.01, and 10 cross-validation folds with the distribution selected as multinomial (Supplementary material 1).

The GBM R package (Ridgeway, 2006) was used to perform prediction analysis using the testing data set and the best fit generated from the training data. GBM uses decision trees in an iterative process to determine the best number of trees leading to the lowest error based on the decline of the loss function. Finally, the GBM package was used to compute the effect values (variable relative influence) for each predictor in the model. The relative influence of each predictor from the tree ensemble is determined as the reduction in the sum of squared error due to the splits on that predictor, then averaging the improvement made by each variable across all the trees to determine the relative effect (Friedman, 2001). Thus, the variables with the largest average decrease in the sum of squared error are considered the most important, expressed as the percentage of the total reduction in the error given by all the predictors. A confusion matrix was developed using the caret package in R that computed the overall statistics, the specific statistics by class, the receiver operating characteristic curve (ROC), and the area under the ROC curve (AUC) values for the multi-class classification to measure the quality of the model's predictions.

For the FUM model, we used the following flags on the training data set: a threshold of 500 trees, interaction depth of 1, shrinkage of 0.01, 10 cross-validation folds, and the distribution was selected as multinomial (Supplementary material 1). The remaining analysis was performed as described for AFL.

### 2.7. Neural network analysis

NN was selected as a second modeling method because of its high performance in the prediction of rare events (Zamani and Kremer, 2013; Gibson and Kroese, 2022). For performing NN training, we first removed the IL county identifier and the year from the data set to reduce potential overfitting. Then, we selected only the input variables that show a relative influence higher than zero in the GBM and then removed the data for the year that was used for validation. Data without the validation set were balanced using the SMOTE method described for GBM. Data were partitioned to create training and testing sets using a 70-to-30 ratio (Supplementary material 1). The same mean and standard deviation scaling was applied to the testing and validation data sets. We trained the NN model in AFL and FUM separately. The best number of hidden layers and neurons was determined by an exhaustive iterative process that aimed to find the combination of NN parameters with the highest accuracy.

### 2.8. Validation analysis using GBM and NN

To perform prediction analysis using the GBM and NN models, we used a single year: 2010 for AFL and 2016 for FUM. These years were selected because their incidence of high contamination events was higher than 3% (more than three high contamination events), and the incidence of high contamination events was 4 and 8.1%, respectively. Validation was performed by using the best fit of GBM and the best number of hidden layers (2 layers) and neurons (65 neurons in layer 1 and 15 neurons in layer 2) for AFL and FUM (65 neurons in layer 1 and 30 neurons in layer 2) determined from the training data (Supplementary material 1). All the input features for the validation years were prepared as previously described in the Methods section for the training data. The mycotoxin data for the years 2010 and 2016 included 99 counties and a total of 99 observations.

R code: Available in Supplementary material 1.

## 3. Results

### 3.1. AFL and FUM contamination in IL

The AFL data showed that only 3.1% of the samples had concentrations >20 ppb, and only 5.4% of the samples had FUM concentrations >5 ppm (Table 1).

**Table 1:** Incidence of AFL and FUM contamination (high or low) in corn in Illinois from 2003 to 2021.

**Year**	**AFL**			**FUM**			**N total**
	**Level**	**Number of cases**	**Incidence**	**Level**	**Number of cases**	**Incidence**	
2003	High	2	2%	High	16	16.20%	99
	Low	97	98%	Low	83	83.80%	
2004	High	0	0%	High	2	2%	99
	Low	99	100%	Low	97	98%	
2008	High	2	2%	High	25	25.30%	99
	Low	97	98%	Low	74	74.70%	
2009	High	0	0%	High	6	6.10%	99
	Low	99	100%	Low	93	93.90%	
2010	High	4	4%	High	0	0%	99
	Low	95	96%	Low	99	100%	
2011	High	2	2%	High	4	4%	99
	Low	97	98%	Low	95	96%	
2012	High	35	35.40%	High	3	3%	99
	Low	64	64.60%	Low	96	97%	
2013	High	2	2%	High	1	1%	99
	Low	97	98%	Low	98	99%	
2014	High	0	0%	High	1	1%	99
	Low	99	100%	Low	98	99%	
2015	High	0	0%	High	0	0%	99
	Low	99	100%	Low	99	100%	
2016	High	1	1%	High	8	8.10%	99
	Low	98	99%	Low	91	91.90%	
2017	High	4	4%	High	1	1%	99
	Low	95	96%	Low	98	99%	
2018	High	2	2%	High	16	16.20%	99
	Low	97	98%	Low	83	83.80%	
2019	High	0	0%	High	3	3%	99
	Low	99	100%	Low	96	97%	
2021	High	1	0.30%	High	10	2.60%	386
	Low	385	99.70%	Low	376	97.40%	

Data were not collected in 2020 due to COVID-19. For AFL high is ≥20 ppb, and for FUM high is ≥5 ppm. N is the number of samples per year.

### 3.2. Weather variables and feature engineering

**Figure 1:**
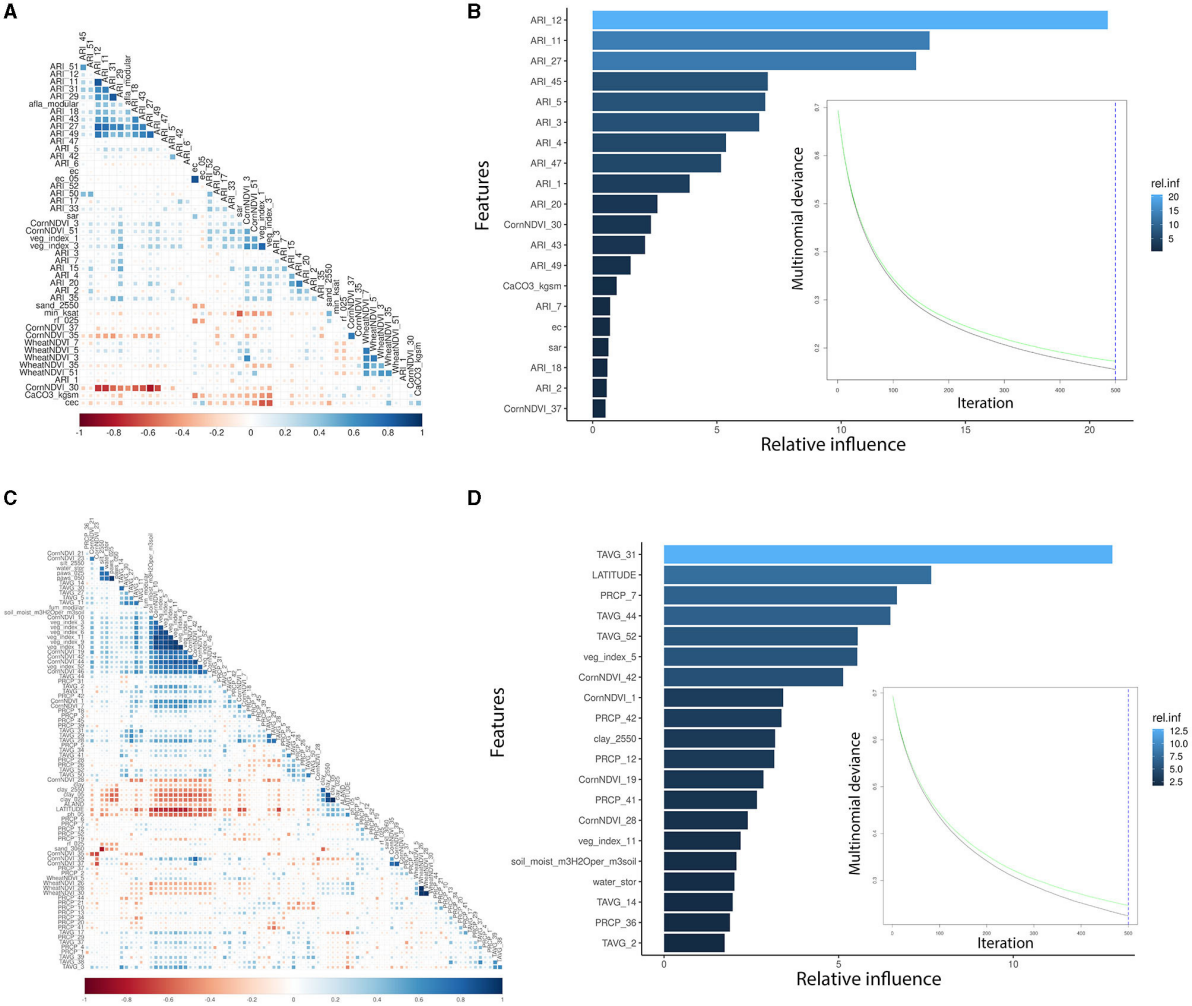
Pair-wise correlation analysis of the significant input variables influencing GBM and summary of the GBM model using multinomial mycotoxin outcome. **(A)** Correlation analysis of the features used for AFL modeling; **(B)** top 20 influential input features and their relative influence over the model in the prediction of AFL; **(C)** correlation analysis of the features used for FUM modeling; **(D)** top 20 influential input features and their relative influence over the model in the prediction of FUM. The correlation is depicted from positive (blue) to negative (red), with blank squares representing non-significant *p*-values of correlation between variables. For the correlation analysis, the *p*-value cutoff was 0.05, and the confidence level was 0.95. The blue hue in GBM plots represents the relative influence of the input variables, with light blue high and dark blue low influence levels. The green line is the testing set multinomial deviance loss, the black line is the training set multinomial deviance loss, and the blue dotted vertical line is the number of iterations used.

AFL risk indexes (ARIs) were the main features engineered by linking fungal growth (Battilani et al., 2013; Van der Fels-Klerx et al., 2019; Liu et al., 2021; Castano-Duque et al., 2022) with the weather. Using ARI instead of temperature and precipitation led to a lower number of input variables incorporated into the AFL model, which also decreased the correlation levels among meteorological variables (Figures 1A, C). For the FUM modeling, we could not use feature engineering functions such as the ARI calculated for AFL produced by *Aspergillus*. FUM output variables showed a significant correlation with general veg_index, corn-specific NDVI, temperature, and several of the soil properties (Figure 1C). Using GBM and only the relative influential variables for the NN led to the inclusion of a lower number of input variables in the models, decreasing the autocorrelation among variables. Furthermore, GBM allows for the control of overfitting caused by high correlation levels among variables by sequentially generating model ensembles that can learn from the errors of previous ensembles (Cooper, 1990; Friedman, 2001).

### 3.3. GBM analysis for AFL and FUM

We used GBM to model AFL and FUM contamination levels (factorial output variable) with our engineered features for AFL and without engineered features for FUM. The models were used to make predictions for the samples in the testing data set (Table 2, Supplementary Table 1). The optimal number of trees for GBM-AFL and GBM-FUM used was 500, which represents the number of trees at which the cross-validation error is minimized (Figures 1B, D). The McNemar *P*-value for the GBM-AFL was 0.4862 and for the GBM-FUM was 0.052 (Supplementary Table 1), which indicated that the proportion of type I and type II errors is the same, meaning that there is homogeneity in the proportion of misclassification. This was expected because the training and testing data sets were balanced by using SMOTE. The overall accuracy of the GBM-AFL model in the test data set (30% of data) was 96%, with a balanced accuracy for high contamination of 96%. For GBM-FUM, the overall accuracy of the test data set was 92%, with a balanced accuracy of 92% (Table 3, Supplementary Table 1). Balanced accuracy or performance is a metric that uses the average of the sensitivity and specificity of the model by considering the predicted classes (high and low contamination). The model performance was the same for the prediction of high and low contamination events. Thus, the balanced accuracy is equal to the overall accuracy. The multi-class area under the curve for GBM-AFL in the testing data set was 0.97 and for GBM-FUM was 0.92, which represents the classifier in its ability to distinguish among classes.

**Table 2:** Contingency tables for GBM-AFL, GBM-FUM, NN-AFL, and NN-FUM from the test (30% of the data) and validation (single year) data sets.

**Model**	**Date set**	**Prediction**	**Reference**	
			**High**	**Low**
GBM-AFL	Test	High	455	14
		Low	19	472
	Validation	High	0	0
		Low	4	95
GBM-FUM	Test	High	445	48
		Low	30	427
	Validation	High	6	20
		Low	2	71
NN-AFL	Test	High	472	2
		Low	14	472
	Validation	High	2	4
		Low	2	91
NN-FUM	Test	High	449	12
		Low	26	463
	Validation	High	8	27
		Low	0	64

**Table 3:** Summary statistics of GBM and NN for AFL and FUM.

	**Overall accuracy test-set**	**Balanced accuracy test-set**	**Overall accuracy validation-set (single year)**	**Balanced accuracy validation-set (single year)**
AFL-GBM	96%	96%	96%	50%
AFL-NN	98%^*^ (46/25/15/2)^**^	98%^*^	94%^*^	73%^*^
FUM-GBM	92%	92%	78%	76%
FUM-NN	96%^*^ (80/65/30/2)^**^	96%^*^	73%^*^	85%^*^

Overall and balanced accuracy for AFL used 2010 as the independent year and FUM used 2016 (Year was not used as an input variable).

* Used only input variables with non-zero influence in the GBM.

** Number of input variables/number of neurons in the first hidden layer/number of neurons in the second hidden layer/number of outputs.

**Figure 2:**
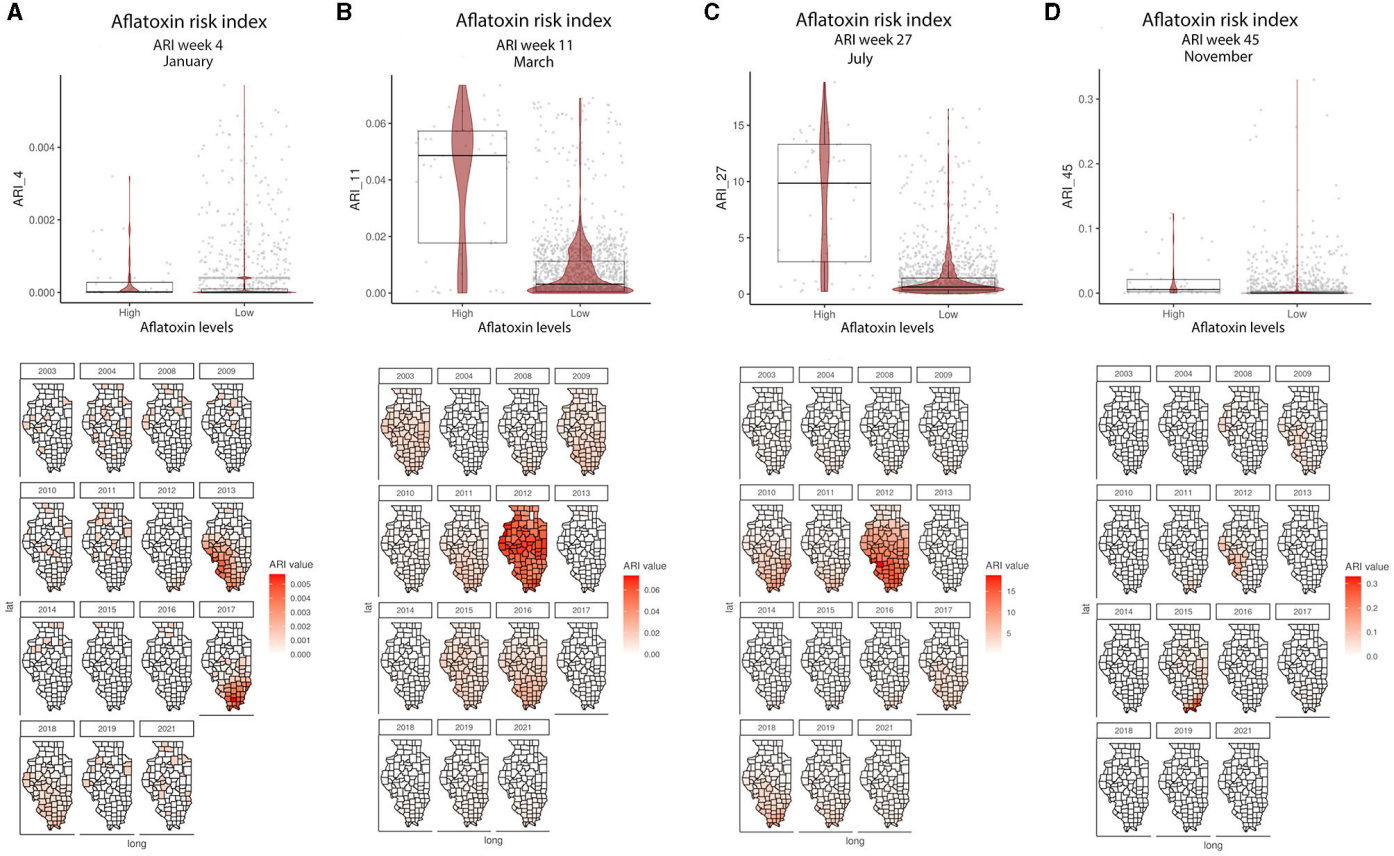
ARI relationship with AFL contamination levels and their geospatial distribution in IL from 2003 to 2021. **(A)** Average ARI in week 4 (January); **(B)** average ARI in week 11 (March); **(C)** average ARI in week 27 (July); **(D)** average ARI 45 (November). Box–Whiskers plot depicts the maximum (25th – 1.5 * interquartile range “IQR”) and minimum [75th percentile + 1.5 * interquartile range (IQR)], and the Box–Whisker plot depicts median, first (25th percentile) and third (75th percentile) quantiles distribution; For AFL, high is >20 ppb and low ≤20 ppb. The violin plot is shaded in red and depicts the density distribution of the soil property in low and high levels of mycotoxin contamination; and the gray dots depict each data point. Maps are shaded in red in relation to the ARI value for a specific week in each year; and the y-axis is latitude and the x-axis is longitude.

The GBM-AFL model showed that of the 153 input features (predictors), only 46 had non-zero influence (Supplementary Table 1). The GBM-FUM showed that of the 199 input features, only 85 had non-zero influence (Supplementary Table 1). For the GBM-AFL model, the top 20 influential features were: ARI in January (Weeks 1, 2, 3, and 4), in February (Weeks 5 and 7), in March (Weeks 11 and 12), in April (Week 18), in May (Week 20), in July (Weeks 27 and 30), in September (Week 37), in October (Week 43), in November (Weeks 45 and 47), and in December (Week 49), soil calcium carbonate (CaCO3), soil electrical conductivity (ec) and soil sodium absorption ratio (sar) (Figure 2, Supplementary Table 1).

**Figure 3:**
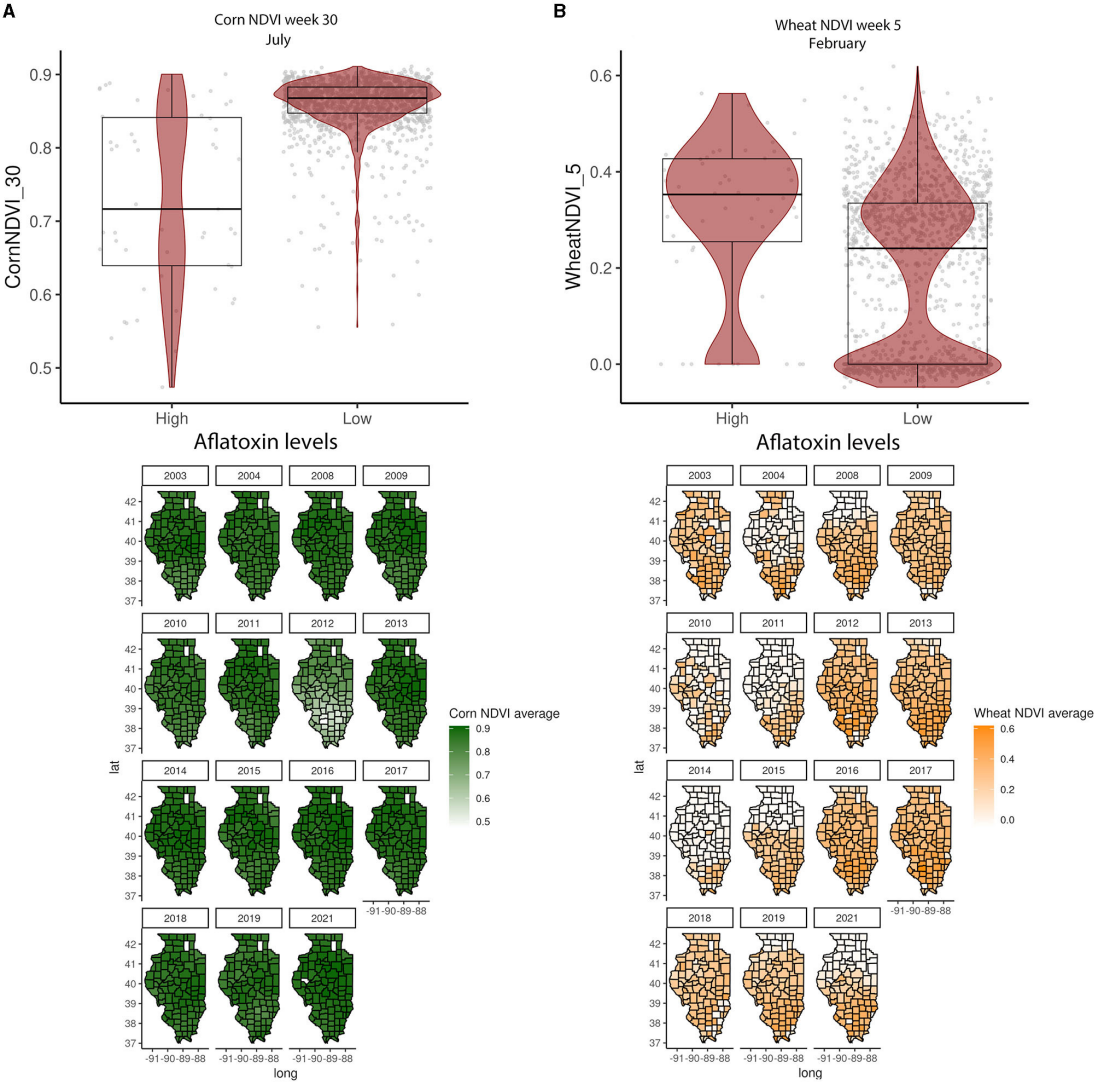
Crop-specific NDVI relationship with AFL contamination levels and their spatial distribution in Illinois from 2003 to 2021. **(A)** Corn NDVI in week 30 (July); **(B)** wheat NDVI in week 5 (February). Box–Whisker plot depicts the maximum (25th – 1.5 * interquartile range “IQR”) and minimum [75th percentile + 1.5 * interquartile range (IQR)], and the Box–Whisker plot depicts median, first (25th percentile), and third (75th percentile) quantiles distribution. For AFL, high is >20 ppb and low ≤20 ppb. The violin plot is shaded in red and depicts the density distribution of the soil property in low and high levels of mycotoxin contamination. The gray dots depict each data point. Maps show the average NDVI values for corn (green) and wheat (yellow) for specific weeks each year.

**Figure 4:**
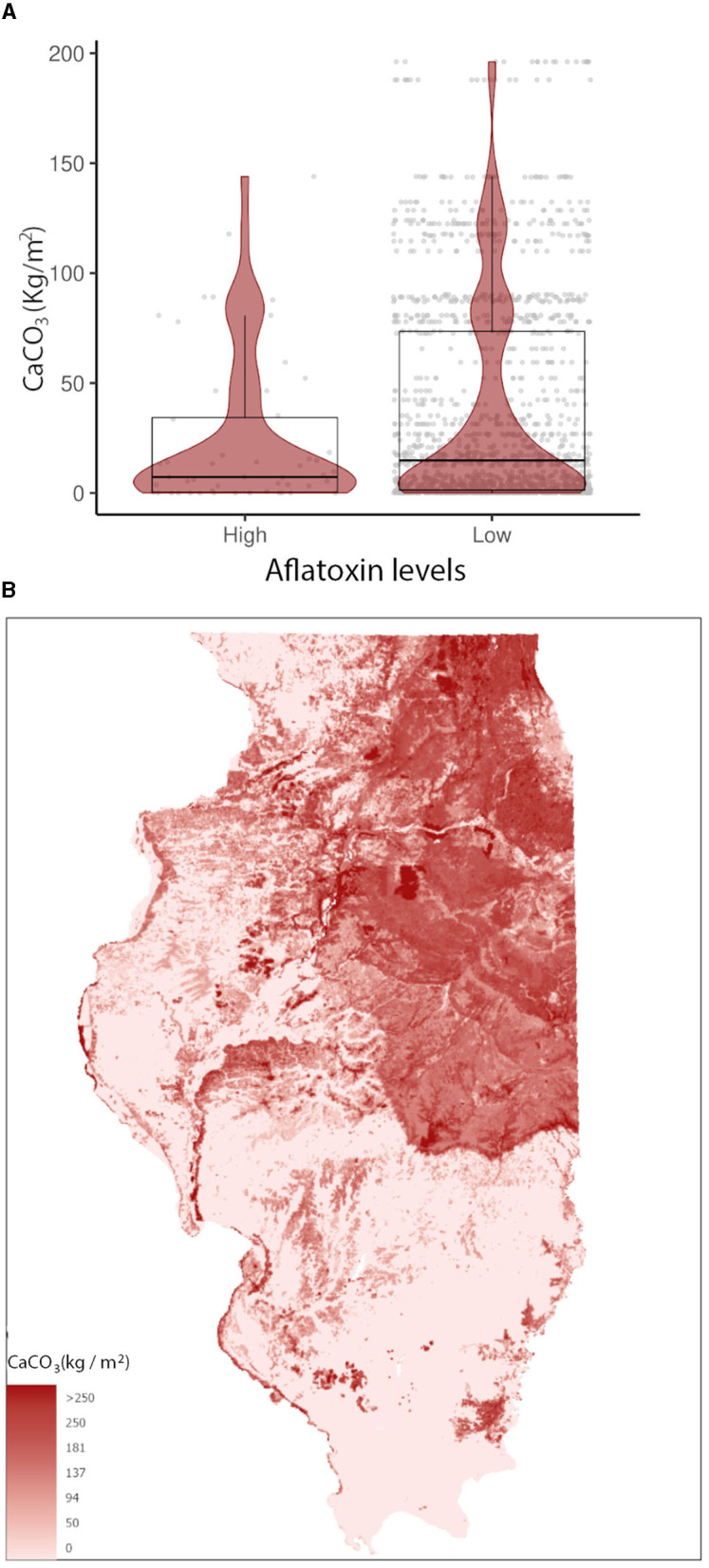
Calcium carbonate (CaCO_3_ Kg/m^2^) relationship with **(A)** AFL contamination levels from 2003 to 2021 and its **(B)** spatial distribution in IL. Box–Whisker plot depicts the maximum (25th – 1.5 * interquartile range “IQR”) and minimum [75th percentile + 1.5 * interquartile range (IQR)], and the Box–Whisker plot depicts median, first (25th percentile), and third (75th percentile) quantiles distribution. For AFL, high is >20 ppb and low ≤20 ppb. The violin plot is shaded in red and depicts the density distribution of calcium carbonate in low and elevated levels of mycotoxin contamination; and the gray dots depict each data point. Maps show the weight percentage of calcium carbonate values. For color legend, a non-linear ramp was used.

In the GBM-AFL, ARI showed a positive correlation with AFL contamination, meaning that high ARI led to high toxin contamination (Figures 1A, 2). At a geospatial level, the ARI throughout IL tends to be higher in the southern region except during 2012, the year with the highest incidence of AFL contamination in the historical data set evaluated (35.4%, Table 1), when the ARI levels were the highest throughout the state in March (week 11) and July (week 27) (Figures 1A, 2). Corn NDVI showed a negative correlation with AFL contamination levels in July (week 30) (Figure 3A) and a positive correlation with wheat NDVI values in February (week 5) (Figure 3B). At a geospatial historical scale, corn NDVI values in week 30 were lower in the southern part of IL in 2012 compared to other years (Figure 3A), and PRCP between weeks 28 and 31 was lower in 2012 compared to other years throughout IL. Calcium carbonate levels in the soil had a negative correlation with AFL levels, meaning that low calcium carbonate tends to be associated with high AFL at harvest (Figure 4A). Lower deposits of calcium carbonate were observed in the southern regions of IL compared to the northeast (Figure 4B). To summarize, the GBM-AFL model showed that ARI pre-planting months (January, February, March, and April) had a strong influence over predictions for AFL contamination at harvest time, and calcium carbonate levels in the soil influence aflatoxin levels.

**Figure 5:**
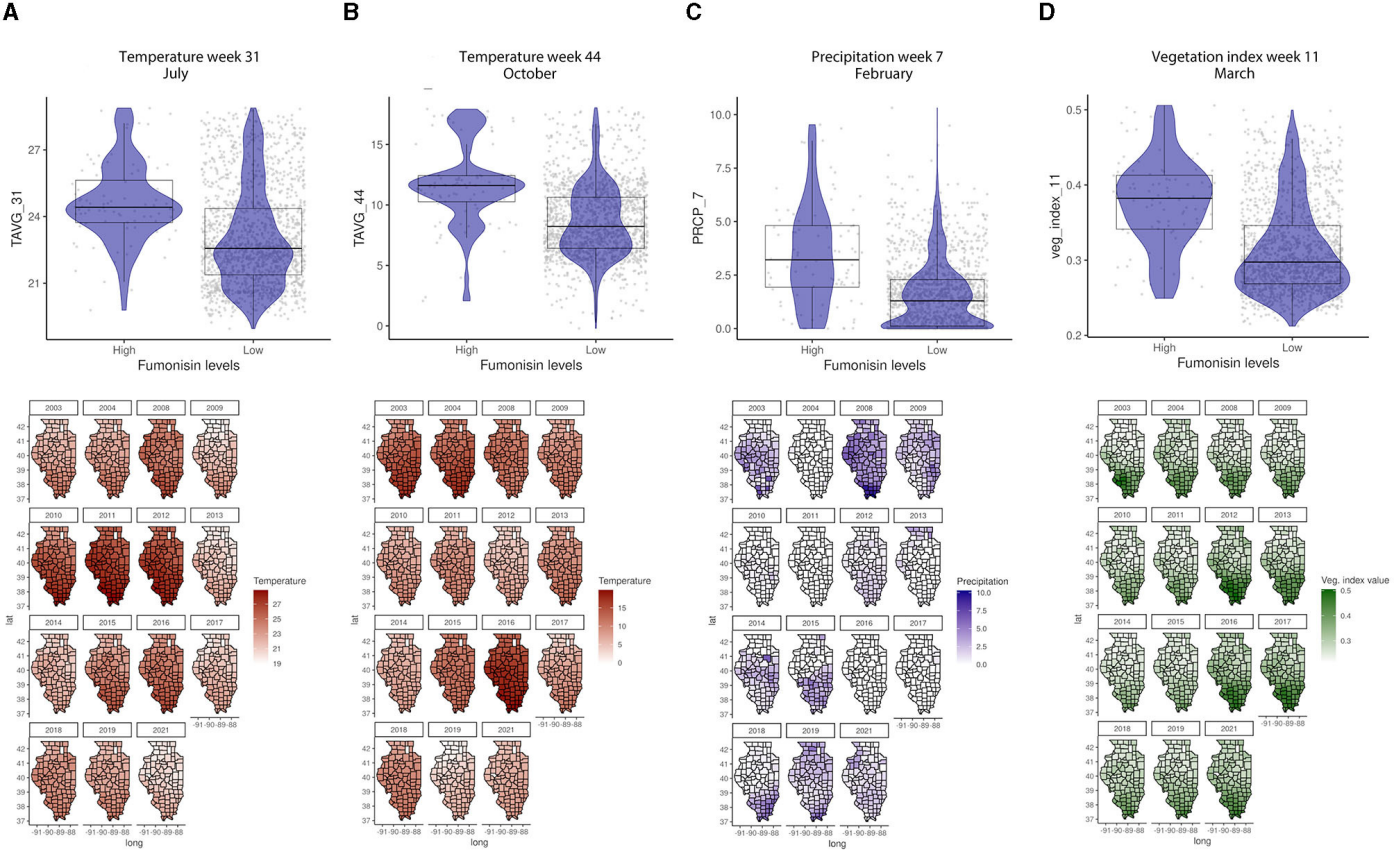
Temperature, precipitation, and veg. index relationships with FUM contamination levels and their geospatial distribution in IL from 2003 to 2021. **(A)** Average temperature in week 31, July, **(B)** average temperature in week 44, October, **(C)** average precipitation in week 7, February, **(D)** average veg. index in week 11, March. Box–Whisker plot depicts the maximum (25th – 1.5 * interquartile range “IQR”) and minimum [75th percentile + 1.5 * interquartile range (IQR)], and the Box–Whisker plot depicts median, first (25th percentile) and third (75th percentile) quantiles distribution; For FUM, high is >5 ppm, and low ≤5 ppm. The violin plot is shaded in blue and depicts the density distribution of the temperature, precipitation, or veg. index in low and high levels of mycotoxin contamination; and the gray dots depict each data point. Maps of geography are shaded in relation to temperature (red), precipitation (blue), or vegetation index (green) values for specific weeks each year, the y-axis is latitude and the x-axis is longitude.

The GBM-FUM showed that of the 199 input features (predictors), only 85 had non-zero influence (Table 2, Supplementary Table 1). Among the 85 features, the top 20 were: latitude; temperature in January (week 2), February (week 5), April (week 14), July (weeks 30 and 31), August (week 34), November/December (week 44), and December (week 52); precipitation in February (week 7), March (week 12), September (weeks 36 and 39), and October (weeks 41 and 42); veg_index February (week 5) and March (week 11); corn-specific NDVI January (week 1), May (week 19), July (week 28), and October (week 42); wheat-specific NDVI in June/July (week 26); clay percentage 25–50 cm depth; water storage and soil water capacity (Supplementary Table 1). In the GBM-FUM, temperature in July (Week 31) and October (Week 44) had a positive relationship with FUM contamination levels (Figures 5A, B), similarly for PRCP in February (Week 7), higher precipitation led to higher FUM contamination levels (Figure 5C) at a geospatial historical level, and high PCRP in March throughout IL in 2008 was linked to the highest incidence of FUM (25.6%, Table 1). To summarize, weather patterns such as precipitation levels in week 7 (February) above 2.5 mm and week 11 (March) above 0.3 mm can lead to higher FUM levels (Figure 5). Furthermore, temperatures in week 31 (July) above 24°C and week 44 (October) above 10°C correlated with high levels of FUM contamination in corn (Figure 5).

**Figure 6:**
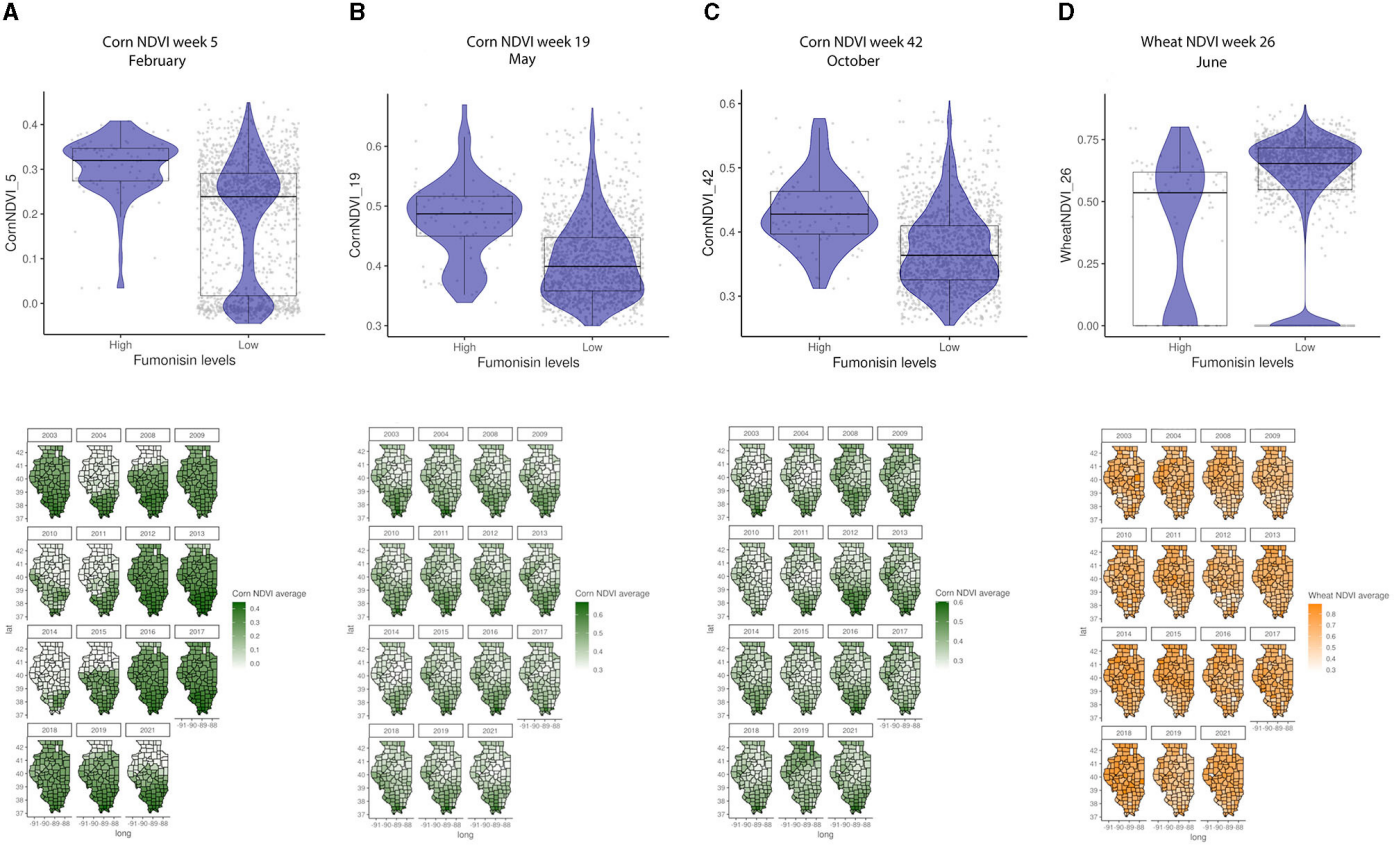
Crop-specific NDVI relationship with FUM contamination levels and their geospatial distribution in IL from 2003 to 2021. **(A)** Corn NDVI in week 5, February, **(B)** corn NDVI in week 19, May, **(C)** corn NDVI in week 42, October, **(D)** wheat NDVI in week 26, June. Box–Whisker plot depicts the maximum (25th – 1.5 * interquartile range “IQR”) and minimum [75th percentile + 1.5 * interquartile range (IQR)], and the Box–Whisker plot depicts median, first (25th percentile) and third (75th percentile) quantiles distribution; For FUM, high is >5 ppm, and low ≤5 ppm. The violin plot is shaded in blue and depicts the density distribution of the corn or wheat NDVI in low and high levels of mycotoxin contamination; and gray dots depict each data point. Maps of geography are shaded in relation to corn NDVI (green) or wheat NDVI (yellow) values for specific weeks in each year.

**Figure 7:**
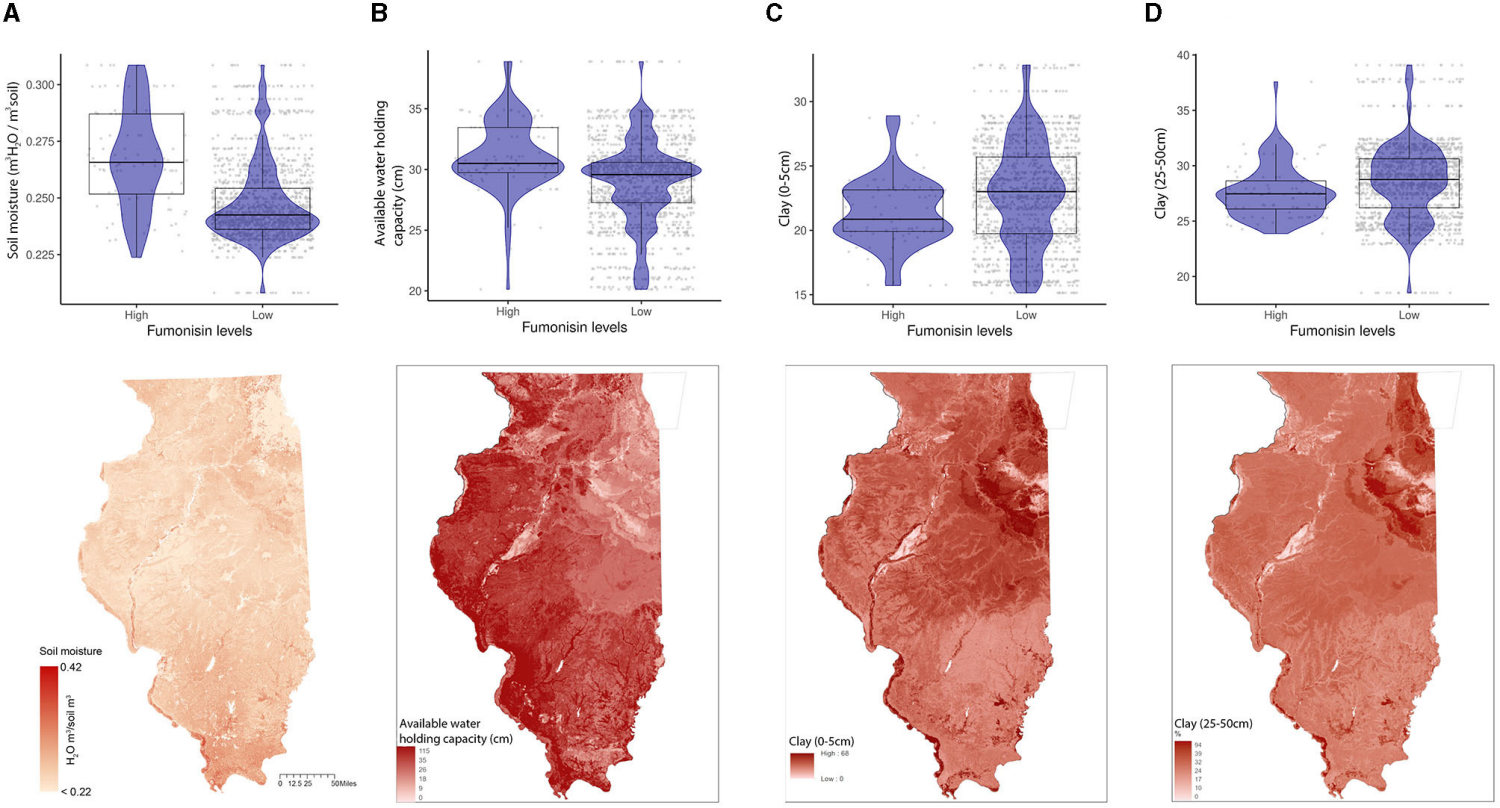
Soil properties relationship with FUM contamination levels and their geospatial distribution in IL. **(A)** soil moisture (m^3^ H2O/m^3^ soil), **(B)** available water-holding capacity (cm). Box–Whisker plot depicts the maximum (25th – 1.5 * interquartile range “IQR”) and minimum [75th percentile + 1.5 * interquartile range (IQR)], and the Box–Whisker plot depicts median, first (25th percentile) and third (75th percentile) quantiles distribution; **(C)** percentage of clay content from 0 to 5 cm below surface, **(D)** percentage of clay content from 25 to 50 cm below surface; For FUM, high is >5 ppm, and low ≤5 ppm. The violin plot is shaded in blue and depicts the density distribution of the soil properties in low and elevated levels of mycotoxin contamination; and the gray dots depict each data point. Maps of geographical shaded red in relation to soil properties values, scale is in miles. For color, legend of soil moisture, a linear ramp was used, and for available water-holding capacity, and clay content, and a non-linear ramp was used.

General vegetation index in March (Week 11) and corn-specific NDVI in February (week 5), May (week 19), and October (week 42) were positively linked to high FUM contamination, which tends to be observed in the southern parts of IL (Figure 6). While the wheat-specific NDVI showed a negative relationship with FUM contamination levels in June (week 26) (Figure 6D). Soil moisture and available water-holding capacity showed a positive correlation with FUM contamination levels, while clay at 0–5 and 25–50 cm showed a negative correlation (Figure 7). At a geospatial scale, both soil moisture and water-holding capacity tend to be higher in the southwest areas of IL, while the clay content tends to be higher in the northeast regions of the state (Figure 7). GBM-FUM showed that pre-planting precipitation and temperature influence the prediction of FUM contamination levels. Furthermore, crop-specific NDVI is a high influencer, as are several soil characteristics linked to water present in the soil.

### 3.4. NN analysis for AFL and FUM

We used NN to model AFL and FUM contamination levels (factorial output variable) by using the input features with non-zero relative influence values from the GBM results (Supplementary Table 1). The McNemar *P*-value for testing NN-AFL was 0.007 and for testing NN-FUM was 0.035 (Supplementary Table 1), which indicated that the proportion of type I and type II errors is not the same, meaning that there is a disproportion of misclassification. This misclassification disproportionality can be seen in the NN-AFL model, which classified 14 data points of 486 as low when they were high and 2 data points of 474 as high when they were low contamination levels (Table 2, Supplementary Table 1). Similarly, the NN-FUM classified 26 data points as low when they were high and 12 as high when they were low (Table 2, Supplementary Table 1). The overall accuracy of the NN-AFL model was 98%, with a balanced accuracy for high contamination of 98%. For NN-FUM, the overall accuracy was 96% and the balanced accuracy was 96% (Table 3, Supplementary Table 1). The multi-class area under the curve for NN-AFL in the testing data set was 0.9835, and for NN-FUM, it was 0.96.

### 3.5. GBM and NN model validation

Model validation was performed by using mycotoxin data from 2010 for AFL (4% incidence) and 2016 for FUM (8.1% incidence) (Table 1). The data included 99 counties and a total of 99 observations in each year (except 2021 with 386 observations). The GBM-AFL model successfully explained low contamination levels of AFL with an accuracy of 96% (Table 3, Supplementary Table 1). The model was not able to correctly predict high contamination events of AFL from the validation data set, which had four observations labeled as high AFL. The overall accuracy of GBM-FUM was 78%, with a specificity of 75% and a balanced accuracy for high levels of 76% (Table 3, Supplementary Table 1). The NN models showed higher balanced accuracy for both AFL and FUM. The NN-AFL model explained high contamination levels with an overall accuracy of 94% and a balanced accuracy of 73% (Table 3, Supplementary Table 1). The NN-AFL model correctly classified 2 out of 4 high contamination events in the validation data set, and only 4 out of 95 data points were misclassified as high when they were low. The McNemar *P*-value for validating NN-AFL was 0.68 (Supplementary Table 1). The NN-FUM model explained high contamination levels with an overall accuracy of 73% and a balanced accuracy of 85% (Table 3, Supplementary Table 1). The NN-FUM model successfully classified all the high contamination events in the validation data set, but it had 27 data points misclassified as high when they were low. The McNemar *P-*value for validating NN-FUM was close to zero (0.0000005, Supplementary Table 1).

## 4. Discussion

Mycotoxin contamination in the US is a pervasive problem that negatively impacts human and animal health and causes losses in the agriculture industry (Vardon et al., 2003; Wu, 2006; Mitchell et al., 2016; Winter and Pereg, 2019). AFL and FUM predictive modeling has been recently studied in the US using machine learning tools (Castano-Duque et al., 2022) that linked weather parameters and latitudinal location in the IL state with historical contamination events of these two mycotoxins. This study builds upon this initial model (Castano-Duque et al., 2022), which provided an IL state case study to account for daily changes in historical weather data (instead of monthly). We also added geospatial soil mineralogical chemical/physical properties and land usage parameters to the models for AFL and FUM. Similarly, predictive mycotoxin models have been developed for northern Italy, linking cropping system factors, and weather variables with deep neural network (DNN) models (Leggieri et al., 2021). Our NN models are unique because we included geospatial dynamic soil and land features linked to the GPS center coordinates of the IL counties. Land usage and soil mineralogical physical/chemical properties are variable for the specific geospatial locations in the US, at both state and county specificity levels (Smith et al., 2019). Using dynamic geospatial data in our models led to the identification of new insights into factors that influence mycotoxin contamination. These factors could be used for risk assessment and to implement timely and region-specific mycotoxin control management practices.

GBM-AFL identified that higher ARI in January, March, July, and November corresponds to higher AFL contamination, meaning that pre-sowing and post-harvest environmental conditions have a strong influence on AFL levels at harvest time. Given that the harvest takes place prior to November, our model assumes that post-harvest input features will influence next year's mycotoxin contamination. Similar correlations were previously proposed by research conducted in the US (Castano-Duque et al., 2022), Africa (Keller et al., 2022), and Serbia (Liu et al., 2021). GBM-FUM showed that high precipitation events during February and March (pre-sowing time), as well as elevated temperatures in July (flowering time) and October (harvest time), influence FUM contamination. Consistently, previous model reports have shown that warmer temperatures early in the planting season (January–April) lead to higher AFL contamination levels in the US (Yu et al., 2022). GBM-AFL showed that corn-specific NDVI at week 30 (July—within flowering time) also influenced AFL contamination; this feature is linked to plant health as NDVI represents a composite satellite measurement of plant greenness. Lower NDVI translates into less green, unhealthy and/or lower density plants, and lower NDVI in week 30 would suggest that the plants were stressed possibly due to abiotic and/or biotic factors.

Previous studies have suggested that crop stress in general also correlates with mycotoxin contamination (Kebede et al., 2012). In 2012, when corn NDVI at week 30 was lower than in other years, the corn crops suffered from extreme drought conditions (NOAA, 2013), which were reflected in lower NDVI values and likely contributed to AFL (contamination levels at harvest time). Studies conducted in Kenya found that AFL contamination is positively associated with high rainfall and NDVI during pre-flowering time while negatively associated during post-flowering time and at harvest (Smith et al., 2016). Our findings agree with published logistic regression models that show that drought stress in corn during flowering time (prior to and beyond mid-silk) is critical for higher contamination at harvest (Damianidis et al., 2018). US-IL models have shown that weather and ARI between corn-growing seasons significantly influence the contamination at the end (Castano-Duque et al., 2022). It is possible that environmental conditions (weather conditions or other agricultural practices) during the spring favor fungal growth (Borràs-Vallverdú et al., 2022), leading to higher corn contamination at the end of the growing season. The development of pest management strategies that take into consideration climate change, such as warmer and wetter spring seasons and drought during the flowering time, is relevant for the management of mycotoxins.

AFL and FUM-GBMs showed that soil properties are also influential factors contributing to the mycotoxin contamination of corn across spatiotemporal scales. Higher calcium carbonate content patterns throughout IL soil were negatively correlated with historical AFL contamination levels. It has been demonstrated that the calcium carbonate content in soils influences soil pH (Weil and Brady, 2002), whereby greater dissolved calcium increases buffering capacity and generates more alkaline soils. Geospatial differences in calcium carbonate in IL soils are due to the advance and retreat of the Laurentide glacier which deposits carbonate-rich materials in the northern half of the state. The negative correlation between AFL levels and calcium carbonate suggests that regions with lower calcium carbonate are associated with higher AFL contamination (Chang and Lynd, 1970), and therefore, calcium carbonate is an important factor in AFL contamination risk.

The abundance of calcium is modulated by both abiotic and biotic factors. In terms of abiotic processes, soils with greater concentrations of CaCO_3_, such as Northern Illinois, typically exhibit greater Ca^2+^ concentrations through the dissolution of parent CaCO_3_ and other Ca-bearing mineralogy via infiltrating rainwater (Smith et al., 2019). In terms of biotic factors, such as soil bacteria, they can also modulate the calcium carbonate content of soil by dissolving calcium carbonate into Ca2+ ions and other carbonate species. Some of these “Calcite Dissolving Bacteria” are *Serratia marcescens, Sphingomonas changbaiensis*, or *Novosphingobium panipatense* (Peper et al., 2022). Furthermore, the soil microbiome can become dominated by microbial assemblages that suppress the growth of AFL-producing fungi with increasing Ca2+ (Zhang et al., 2021), FUM-producing fungi (Srinivasan et al., 2009), and suppression of mycotoxin-producing fungi strains by non-toxin-producing fungi (Mehl et al., 2012; Ehrlich, 2014). The presence of both growth-suppressive bacteria and fungi that dissolve calcium carbonate would potentially lead to positive feedback in a calcium carbonate-rich soil. Fungi are involved in carbonate rock destruction, dissolution, precipitation (Verrecchia et al., 2003), and biomineralization (Bindschedler et al., 2016) in laboratory environments. Other environmental factors, such as wet and dry periods, affect the precipitation and accumulation of calcium carbonate in soil (Birkeland, 1974). It is possible that fungal communities and weather parameters co-influence levels of calcium carbonate and calcium ions in the soil, and these compounds can act as a feedback loop that modulates the fungal communities present in the soil. Understanding the native microbial communities and mycotoxin fungi/contamination could aid in understanding the interaction of soil microbiome, calcium carbonate, and AFL contamination. This knowledge of microbiome diversity could be useful for the development of region-specific biocontrol strategies.

The addition of calcium carbonate in the form of limestone to soils is a major agricultural practice in humid climates worldwide, used to directly change the chemical makeup of the rooting zone and improve soil fertility (Weil and Brady, 2002). Greenhouse experiments with peanut plants (*Arachis hypogaea* L.) showed that adding calcium to the soil leads to a significant reduction in AFL contamination of seeds (Uppala, 2011). Calcium-deficient soils cause calcium deficiency in the corn plant, which can lead to the failure of the upper leaves to unwrap at tasseling time (Melsted, 1953), shorter roots, chlorosis, and necrosis of leaves (do Moraes Gatti et al., 2023). These calcium-deficient symptoms might increase the propensity of fungal infections to thrive, as calcium is an important component of pathogen-associated molecular-triggered immunity (Fountain et al., 2016) and maize defense responses to pathogens (Jiang and Zhang, 2003; Hu et al., 2007; Ma and Berkowitz, 2011). We hypothesize that Northern IL soils, which are richer in CaCO3 and Ca, are conducive to healthier plants capable of greater resistance to fungal disease and supporting microbiomes that also suppress AFL growth. The characterization of IL native microbiome and fungi soil populations linked to their geospatial location in calcium carbonate-rich or poor zones is not known, although this could aid in understanding the interaction of soil microbiome, calcium carbonate, and AFL contamination. This knowledge of microbiome diversity could be useful for the development of region-specific biocontrol strategies and mycotoxin management alongside external limestone application in areas such as Southern IL or other areas with elevated AFL risks, e.g., a corn-growing season with a hot/wet spring followed by drought during flowering time.

GBM-FUM showed that FUM contamination is correlated with soil moisture, available water-holding capacity, and a high percentage of clay. These soil hydropedology properties vary across IL (Smith et al., 2019). Southern IL tends to have higher soil moisture levels compared to the north, with the southern area of the state having historically higher percentages of FUM contamination (Castano-Duque et al., 2022). Correspondingly, a positive correlation was observed between soil moisture and FUM contamination. Other soil properties, such as the percentage of clay, tend to be higher in the northeastern areas of IL (Smith et al., 2019), which historically tend to have lower FUM contamination (Castano-Duque et al., 2022) as demonstrated by the negative correlation between these two variables.

Clay is the smallest mineral component of soil with the highest surface area per unit mass, in general, it has a high water-holding capacity and low hydraulic conductivity compared to other soil textures, making it prone to becoming waterlogged in moist conditions (Libohova et al., 2018), such as heavy rains. Clay's high water-holding capacity and poor permeability to water and gases (Weil and Brady, 2002) can lead to flood stress and waterlogging of plants during high precipitation events. While the relationship between flood-stressed corn and *Fusarium* spp. is not fully understood, it has been established that *Fusarium* is a strongly aerobic fungus (Stover, 1953), and it has been shown that among sandy loam, silt loam, or clay soil types, *Fusarium solani* and *Fusarium tricinctum* have higher disease severity on soybeans grown in sandy and silt loam soil (Yan and Nelson, 2022). Clay soils have high infiltration capacities as water moves into the shrinkage cracks, but when cracks are not present, their infiltration rate is characteristically slow. The small pores of clay solids when filled with water (saturated and under waterlogging conditions) could also influence *Fusarium* spp by limiting oxygen availability. It would be key to predict FUM outbreaks to understand the biological effect of clay-rich soils and high precipitation events on *Fusarium* growth and the diversity of species in different geospatial locations in the US.

Soils in IL with prominent levels of clay, low soil moisture, and low water-holding capacity offer conditions that historically might lead to lower FUM contamination. Clay-rich soils bind strongly to organic matter, contributing to soil aggregation, soil fertility, and cation exchange. Therefore, clay influences soil fertility by modulating cation exchange capacity and soil physical properties (Weil and Brady, 2002). The capacity of clay to hold water and bind to organic matter affects water in the soil and nutrients available for crops. High precipitation events early in the year (February), when there is no corn in the field, could lead to higher FUM contamination. We hypothesize that there must be some biological and chemical interaction between clay-rich soils early in the season and snow and rain events that influence fungal growth in the fields.

The primary abiotic factors contributing to the presence of FUM are water activity and temperature (Sanchis et al., 2006; Picot et al., 2010), but the nature of this relation is complex in the context of soil, hydroclimate variability, and plant water use. In optimal temperatures, increased water activity promotes FUM production, but at temperatures above the thermal optimum of 25°C, decreased water activity also promotes greater FUM production (Marin et al., 2004; Samapundo et al., 2005; Sanchis et al., 2006; Picot et al., 2010). Furthermore, *F. verticillioides* growth has been shown to be limited by low water activity; however, the FUM1 gene expression was significantly greater with lower water activity (Jurado et al., 2008). Therefore, favorable conditions for FUM production may occur under two different hydroclimate conditions: (1) a wet, i.e., high water activity, period with temperatures in the thermal optimum range; and (2) temperatures above the thermal optimum but dry, i.e., low water activity. Some field-based evidence corroborates the latter, where Roucou et al. (2021) noted increased FUM risk with greater water stress, but only when temperatures were elevated. In this study, the greater historical occurrence of FUM in Southern IL, where soils have less water and a propensity for water stress, would support the second condition. Another biophysical feedback between crop and soil may also drive greater water stress as starches are produced during kernel ripening (Jurado et al., 2008; Picot et al., 2010). As such, positive feedback driving greater risk for FUM production into the latter part of a growing season may develop when: (1) temperatures are elevated; (2) kernel ripening begins and plant water use increases; (3) water stress is persistent, e.g., drought-like conditions; and (4) crops are grown in soils that exasperate water-holding capacity.

Depending on the soil type, complex plant–water interactions can affect plant health positively or negatively. Soils conducive to drought or flooding stress can result in crops with a lower ability to fight fungal infections and increased susceptibility to AFL (Kebede et al., 2012) and FUM (Parsons and Munkvold, 2010; Vaughan et al., 2016) contamination. Furthermore, relative humidity affects fungal sporulation and growth (Foister, 1946); therefore, both soil–fungi–water interactions and plant–fungi–water interactions will affect mycotoxin outbreaks. Further research that includes field trials from different regions of the US that have variable soil types and includes year-round time frames to account for the variation of precipitation and temperature would aid in understanding the causality that these spatial-temporal variables have on fungal load and mycotoxin contamination.

The AFL and FUM-GBM models showed better accuracy values than previously reported in the monthly-based models that did not include geospatial soil dynamic features (Castano-Duque et al., 2022). NN models showed high class-specific performance for 1-year predictive validation for AFL (73%) and FUM (85%), thus the NN models are recommended for annual mycotoxin prediction. The observed overall and specific accuracy levels are at par when compared to other mycotoxin non-US models that showed ranges from 90 to 99% general accuracy for wheat models in Europe (Wang et al., 2022), 75% general accuracy for corn using AFL-maize models in Italy (Battilani et al., 2013) and >75% general accuracy using machine learning models for toxin prediction in corn in northern Italy (Leggieri et al., 2021). Because GBM and NN models showed high-performance levels (Table 3), both can be used for annual mycotoxin prediction. To be implemented, these models would need to use input features for the months of October–December from the previous year's post-harvest and projected weather and NDVI values for a maximum of a month prior to harvest.

Our dynamic geospatial models offer additional information about soil properties and remote-sensed data (NDVI) that influence AFL and FUM contamination in corn. *Aspergillus* growth begins in the soil, which is the habitat for the fungus; thus, the production of AFL by fungi will be influenced by agricultural practices, environmental conditions, and fungal interaction with soil and plants (Winter and Pereg, 2019). Several studies have shown a significant correlation between climate, soil properties, NDVI, and agricultural management practices and AFL contamination in several countries in sub-Saharan Africa, such as Kenya, Zambia, and others, as reviewed by Keller et al. (2022). AFL surveys and studies conducted in Eastern and Western Kenya (Köppen, 2011) support the utility of remote-sensed data such as NDVI, rainfall, and soil properties to predict aflatoxin contamination (Smith et al., 2016). Satellite-generated databases have been previously used in wheat studies in Europe and led to higher model accuracy (Wang et al., 2022). Previous studies in Africa found that soil organic carbon content, pH, total exchangeable bases, salinity, texture, and soil type were significantly associated with AFL (Smith et al., 2016). Soil data throughout IL embody important geographic variability related to geologic deposition, age of material, climate, duration of pedogenesis, natural history, and other factors. Soil conditions strongly influence which species of fungi dominate in a given soil environment (Weil and Brady, 2002). Our research shows that in the US, specific properties available from geospatial data such as calcium carbonate, pH, clay content, and soil moisture are key factors correlated with historical AFL and FUM outbreaks. Our dynamic geospatial models can guide research and identify multiple factors contributing to the risk of AFL or FUM contamination in a geographical region and within weekly time frames.

Some agronomic practices take place between corn-growing seasons, such as tilling and drilling (Borràs-Vallverdú et al., 2022), while other factors are constant throughout the year, such as soil properties (Smith et al., 2016) and other socio-economical parameters such as training farmers on the detection of contaminated crops in the field and mitigation strategies, and the involvement of the private sector to provide incentives for farmers to adopt aflatoxin management techniques (Titilayo, 2018) that influence mycotoxin contamination. Several of these factors, such as soil properties, were included in our models, although much information on other agronomic practices is not available at the historical geospatial scale needed for this mycotoxin data set. Nevertheless, our models were able to determine that input features linked to weekly temporal zones between corn-growing seasons have a significant influence on the end-of-year mycotoxin contamination. Among these input features, soil properties were a novel addition to the IL models. It remains to be evaluated if there are differences between the diverse fungi *Aspergillus* (AFL producer) and *Fusarium* (FUM producer) (Samapundo et al., 2005) in relation to growth, development, and toxin production associated with environmental factors in the field, such as crop rotation using winter wheat. For future applications, quarterly historical data should be used to create a quarterly predictive model. These models would be beneficial for farmers as they will be alerted by the prediction of impending contamination several months in advance before harvest, thus providing ample time to deploy mitigation measures such as biocontrol, irrigation, and/or fungicide application to avoid contamination of their crops.

## Data availability statement

The datasets presented in this study can be found in online repositories. The names of the repository/repositories and accession number(s) can be found below: https://www.gro-intelligence.com/, https://doi.org/10.5067/MODIS/MOD13A2.061, Cropscape: https://nassgeodata.gmu.edu/CropScape/.

## Author contributions

LC-D: Conceptualization, Data curation, Formal analysis, Investigation, Methodology, Supervision, Validation, Visualization, Writing—original draft, Writing—review & editing. EW: Data curation, Methodology, Software, Visualization, Writing—review & editing. JB: Data curation, Methodology, Software, Visualization, Writing—review & editing. CL: Conceptualization, Resources, Software, Supervision, Writing—review & editing. NV: Data curation, Methodology, Writing—review & editing. MF: Conceptualization, Methodology, Resources, Software, Writing—review & editing. KB: Data curation, Investigation, Resources, Writing—review & editing. PO: Project administration, Resources, Supervision, Writing—review & editing. HF-K: Conceptualization, Project administration, Resources, Supervision, Writing—review & editing. MV: Conceptualization, Investigation, Project administration, Resources, Supervision, Writing—review & editing. KR: Conceptualization, Investigation, Project administration, Resources, Supervision, Writing—review & editing.
