# Supplementary material for: Dynamic geospatial modeling of mycotoxin contamination of corn in Illinois: unveiling critical factors and predictive insights with machine learning
Source: Front Microbiol. 2023 Nov 1;14:1283127. doi: 10.3389/fmicb.2023.1283127 (PMC10646420; doi:10.3389/fmicb.2023.1283127)
Supplement: Supplementary file 1 [file Data_Sheet_1.docx]

**sessionInfo()**

R version 4.2.0 (2022-04-22)

Platform: x86_64-pc-linux-gnu (64-bit)

Running under: Ubuntu 20.04.4 LTS

attached base packages:

splines grid stats graphics grDevices utils datasets methods

base

other attached packages:

vtable_1.3.3 kableExtra_1.3.4 naniar_0.6.1

NHANES_2.1.0 mice_3.14.0 VIM_6.1.1

colorspace_2.0-3 pROC_1.18.0 ranger_0.14.1

rpart.plot_3.1.1 rpart_4.1.16 forecast_8.16

quantmod_0.4.20 TTR_0.24.3 xts_0.12.1

zoo_1.8-10 randomForest_4.7-1.1 rattle_5.5.1

bitops_1.0-7 e1071_1.7-11 choroplethrMaps_1.0.1

corrplot_0.92 gridExtra_2.3 ISLR_1.4

Rgraphviz_2.40.0 graph_1.74.0 BiocGenerics_0.42.0

lubridate_1.8.0 forcats_0.5.1 stringr_1.4.0

purrr_0.3.4 readr_2.1.2 tidyr_1.2.0

tibble_3.1.7 tidyverse_1.3.1 ggpubr_0.4.0

dplyr_1.0.9 gbm_2.1.8 RANN_2.6.1

doSNOW_1.0.20 snow_0.4-4 iterators_1.0.14

foreach_1.5.2 caret_6.0-92 visNetwork_2.1.0

bnlearn_4.7.1 igraph_1.3.2 RColorBrewer_1.1-3

lattice_0.20-45 ggplot2_3.3.6 remotes_2.4.2

loaded via a namespace (and not attached):

readxl_1.4.0 backports_1.4.1 systemfonts_1.0.4

plyr_1.8.7 sp_1.5-0 listenv_0.8.0

digest_0.6.29 htmltools_0.5.2 fansi_1.0.3

magrittr_2.0.3 tzdb_0.3.0 recipes_0.2.0

globals_0.15.0 modelr_0.1.8 gower_1.0.0

svglite_2.1.0 hardhat_1.1.0 tseries_0.10-51

rvest_1.0.2 haven_2.5.0 xfun_0.31

crayon_1.5.1 jsonlite_1.8.0 survival_3.3-1

glue_1.6.2 gtable_0.3.0 ipred_0.9-13

webshot_0.5.3 car_3.1-0 future.apply_1.9.0

DEoptimR_1.0-11 abind_1.4-5 scales_1.2.0

DBI_1.1.3 rstatix_0.7.0 Rcpp_1.0.8.3

viridisLite_0.4.0 laeken_0.5.2 proxy_0.4-27

stats4_4.2.0 lava_1.6.10 prodlim_2019.11.13

vcd_1.4-10 htmlwidgets_1.5.4 httr_1.4.3

ellipsis_0.3.2 pkgconfig_2.0.3 nnet_7.3-17

dbplyr_2.2.0 utf8_1.2.2 tidyselect_1.1.2

rlang_1.0.2 reshape2_1.4.4 munsell_0.5.0

cellranger_1.1.0 tools_4.2.0 cli_3.4.1

generics_0.1.2 broom_0.8.0 evaluate_0.15

fastmap_1.1.0 yaml_2.3.5 ModelMetrics_1.2.2.2

knitr_1.39 fs_1.5.2 robustbase_0.95-0

visdat_0.5.3 future_1.26.1 nlme_3.1-157

xml2_1.3.3 compiler_4.2.0 rstudioapi_0.13

curl_4.3.2 ggsignif_0.6.3 reprex_2.0.1

stringi_1.7.6 smotefamily_1.3.1 Matrix_1.4-1

urca_1.3-0 vctrs_0.4.1 pillar_1.7.0

lifecycle_1.0.1 lmtest_0.9-40 data.table_1.14.2

R6_2.5.1 parallelly_1.32.0 codetools_0.2-18

boot_1.3-28 MASS_7.3-56 assertthat_0.2.1

withr_2.5.0 fracdiff_1.5-1 parallel_4.2.0

hms_1.1.1 quadprog_1.5-8 timeDate_3043.102

class_7.3-20 rmarkdown_2.14 carData_3.0-5

set.seed(1234)

*# Create a multinomial variable for both, aflatoxin and fumonisin*

all_data_IL$afla_modular <- ifelse(all_data_IL$Aflatoxin_Result_Avg_.ppb. > 20 , "High", "Low")

all_data_IL$fum_modular <- ifelse(all_data_IL$Fumonisin_Result_Avg_.ppm > 5 , "High", "Low")

*# all_data_IL_AFL data set are the selected input variable for AFL modeling*

*# all_data_IL_FUM data set are the selected input variable for FUM modeling*

*# Create a more balanced data base using SMOTE*

*#154 column is the response variable*

new_afla_IL <-smotefamily::SMOTE(all_data_IL_AFL [,-154], as.numeric(all_data_IL_AFL $afla_modular))

synt_afla_IL<-(new_afla_IL$data)

*#NOTE THAT 1 -> LOW AND 2 -> HIGH*

synt_afla_IL<-synt_afla_IL %>% rename("afla_modular" = "class")

synt_afla_IL$afla_modular[synt_afla_IL$afla_modular == '1'] <- 'High'

synt_afla_IL$afla_modular[synt_afla_IL$afla_modular == '2'] <- 'Low'

*#200 column is the response variable*

new_fum_IL <-smotefamily::SMOTE(all_data_IL_FUM[,-200], as.numeric(all_data_IL_FUM $fum_modular))

synt_fum_IL<-(new_fum_IL$data)

synt_fum_IL<-synt_fum_IL %>% rename("fum_modular" = "class")

synt_fum_IL$fum_modular[synt_fum_IL$fum_modular == '1'] <- 'High'

synt_fum_IL$fum_modular[synt_fum_IL$fum_modular == '2'] <- 'Low'

***#FOR GBM AFL***

tmp1<-as.data.frame(synt_afla_IL)

tmp1$afla_modular<-as.factor(tmp1$afla_modular)

inTrain1<-createDataPartition(y=as.factor(tmp1$afla_modular),

p=0.7, list = FALSE,times = 1)

training1 <- tmp1[inTrain1,]

testing1 <- tmp1[-inTrain1,]

training1$afla_modular<-as.factor(training1$afla_modular)

fit.gbm.1 <- gbm(training1$afla_modular ~ .,

data = select(training1,-afla_modular),

n.trees = 500,

distribution = "multinomial",

interaction.depth = 1,

shrinkage = 0.01,

cv.folds = 10,

n.cores=1)

*#Run the aflatoxin prediction using the gbm model*

preds2021.gbm.1 <- predict(object=fit.gbm.1,

newdata=tmp,

ntree = best.fit.gbm.1,

type='response')

***#FOR GBM FUM***

tmp2<-as.data.frame(synt_fum_IL)

tmp2$fum_modular<-as.factor(tmp2$fum_modular)

inTrain2<-createDataPartition(y=as.factor(tmp2$fum_modular),

p=0.7, list = FALSE,times = 1)

training2 <- tmp2[inTrain2,]

testing2 <- tmp2[-inTrain2,]

training2$fum_modular<-as.factor(training2$fum_modular)

fit.gbm.2 <- gbm(training2$fum_modular ~ .,

data = select(training2,-fum_modular),

n.trees = 500,

distribution = "multinomial",

interaction.depth = 1,

shrinkage = 0.01,

cv.folds = 10,

n.cores=1)

*#Run the fumonisin prediction using the gbm model*

set.seed(1)

preds2021.gbm.2 <- predict(object=fit.gbm.2,

newdata=tmp,

ntree = best.fit.gbm.2,

type='response')

***#FOR NEURAL NETWORK MODELING TAKE THE DATA BASE WITH THE AFL AND FUM IN HIGH AND LOW CATEGORICAL VARIABLES THEN SELECT ONLY THE INPUT VARIABLES THAT SHOW NON-ZERO INFLUENCE LEVELS FROM GBM THE NEW DATA BASES ARE:***

*#afla_IL_GBM FOR AFL DATA*

*#fum_IL_GBM FOR FUM DATA*

***#AFLATOXIN NN Modeling***

*#use SMOTE to create new dataset that is more balanced*

*#column 1 is the response variable*

new_afla_IL <-smotefamily::SMOTE(afla_IL_GBM[,-1], as.numeric(afla_IL_GBM$afla_modular))

synt_afla_IL<-synt_afla_IL %>% dplyr::rename("afla_modular" = "class")

synt_afla_IL$afla_modular[synt_afla_IL$afla_modular == '1'] <- 'High'

synt_afla_IL$afla_modular[synt_afla_IL$afla_modular == '2'] <- 'Low'

*#You need to make sure your columns are all numeric and you got rid of the factor columns*

tmp1<-synt_afla_IL

inTrain1<-createDataPartition(y=(tmp1$afla_modular),

p=0.7, list = FALSE,times = 1)

training1 <- tmp1[inTrain1,]

testing1 <- tmp1[-inTrain1,]

*#column 47 is the output variable*

training1out<-training1[,47]

testing1out<-testing1[,47]

training1<- as.data.frame(lapply(training1[,-47], as.numeric))

testing1<- as.data.frame(lapply(testing1[,-47], as.numeric))

tmp1_num<- as.data.frame(lapply(tmp1[,-47], as.numeric))

m <- colMeans(training1)

s <- apply(training1, 2, sd)

training1 <- training1 %>% scale(center = m, scale = s)

testing1 <- testing1 %>%scale(center = m, scale = s)

validating1 <- validating1 %>% scale(center = m, scale = s)

n <- neuralnet(training1out~.,

data = training1,

hidden = c(25,15),#adjust the hidden layers

err.fct = "ce",

linear.output = FALSE)

outputtesttest <- compute(n, testing1)

outputtestvalidate <- compute(n, validating1)

***#FUMONISIN NN Modeling***

new_fum_IL <-smotefamily::SMOTE(fum_IL_GBM[,-1], as.numeric(fum_IL_GBM$fum_modular))

synt_fum_IL<-synt_fum_IL %>% dplyr::rename("fum_modular" = "class")

synt_fum_IL$fum_modular[synt_fum_IL$fum_modular == '1'] <- 'High'

synt_fum_IL$fum_modular[synt_fum_IL$fum_modular == '2'] <- 'Low'

tmp2<-synt_fum_IL

inTrain2<-createDataPartition(y=(tmp2$fum_modular),

p=0.7, list = FALSE,times = 1)

training2 <- tmp2[inTrain2,]

testing2 <- tmp2[-inTrain2,]

training2out<-training2[,86]

testing2out<-testing2[,86]

training2<- as.data.frame(lapply(training2[,-86], as.numeric))

testing2<- as.data.frame(lapply(testing2[,-86], as.numeric))

m <- colMeans(training2)

s <- apply(training2, 2, sd)

training2 <- training2 %>% scale(center = m, scale = s)

testing2 <- testing2 %>%scale(center = m, scale = s)

validating2 <- validating2 %>% scale(center = m, scale = s)

n <- neuralnet(training2out~.,

data = training2,

hidden = c(65,30),#adjust the hidden layers

err.fct = "ce",

linear.output = FALSE)

outputtest <- compute(n, testing2)

outputvalidate <- compute(n, validating2)
